# One Novel Phantom-Less Quantitative Computed Tomography System for Auto-Diagnosis of Osteoporosis Utilizes Low-Dose Chest Computed Tomography Obtained for COVID-19 Screening

**DOI:** 10.3389/fbioe.2022.856753

**Published:** 2022-06-28

**Authors:** Tang Xiongfeng, Zhang Cheng, He Meng, Ma Chi, Guo Deming, Qi Huan, Chen Bo, Yang Kedi, Shen Xianyue, Wong Tak-Man, Lu William Weijia, Qin Yanguo

**Affiliations:** ^1^ Department of Orthopaedics, The Second Hospital of Jilin University, Changchun, China; ^2^ Department of Orthopaedics and Traumatology, The University of Hong Kong, Hong Kong, Hong Kong SAR, China; ^3^ Department of Orthopaedics and Traumatology, The University of Hong Kong-Shenzhen Hospital, Shenzhen, China; ^4^ Faculty of Pharmaceutical Science, Shenzhen Institute of Advanced Technology, Chinese Academy of Sciences, Shenzhen, China

**Keywords:** osteoporosis, phantom-less QCT, dual-energy X-ray, low-dose CT, COVID-19

## Abstract

**Background:** The diagnosis of osteoporosis is still one of the most critical topics for orthopedic surgeons worldwide. One research direction is to use existing clinical imaging data for accurate measurements of bone mineral density (BMD) without additional radiation.

**Methods:** A novel phantom-less quantitative computed tomography (PL-QCT) system was developed to measure BMD and diagnose osteoporosis, as our previous study reported. Compared with traditional phantom-less QCT, this tool can conduct an automatic selection of body tissues and complete the BMD calibration with high efficacy and precision. The function has great advantages in big data screening and thus expands the scope of use of this novel PL-QCT. In this study, we utilized lung cancer or COVID-19 screening low-dose computed tomography (LDCT) of 649 patients for BMD calibration by the novel PL-QCT, and we made the BMD changes with age based on this PL-QCT.

**Results:** The results show that the novel PL-QCT can predict osteoporosis with relatively high accuracy and precision using LDCT, and the AUC values range from 0.68 to 0.88 with DXA results as diagnosis reference. The relationship between PL-QCT BMD with age is close to the real trend population (from ∼160 mg/cc in less than 30 years old to ∼70 mg/cc in greater than 80 years old for both female and male groups). Additionally, the calculation results of Pearson’s r-values for correlation between CT values with BMD in different CT devices were 0.85–0.99.

**Conclusion:** To our knowledge, it is the first time for automatic PL-QCT to evaluate the performance against dual-energy X-ray absorptiometry (DXA) in LDCT images. The results indicate that it may be a promising tool for individuals screened for low-dose chest computed tomography.

## Introduction

Osteoporosis is a complex disease in which the quantity and quality of bone are diminished, causing an increase in bone fragility (Johnell and Kanis, 2006). Osteoporosis and osteoporotic fractures have become global health issues of major concern with the growth in the aging population (Alejandro and Constantinescu, 2018). About 200 million people suffer from osteoporosis, and 89 million fractures occur worldwide every year, with considerable health, societal, and economic burden (Pisani et al., 2016). The prevalence of osteoporosis and the incidence of fragility fracture in china have increased markedly over the last decades. Recent data report an osteoporosis prevalence of 29.1% in women and 6.5% in men aged >50 years, equating to an estimated population prevalence of 49.3 million and 10.9 million, respectively. Approximately 50% of women will have at least one fracture after the age of 50 years (Reid, 2020). It is estimated that by 2050, there will be 5.99 (95% CI, 5.44–6.55) million fractures annually in China, costing $25.43 (95% CI, $23.92 to $26.95) billion, reflecting a 2.7-fold increase since 2010 (Chen et al., 2016). The increase in osteoporosis and fracture rates reflects in part the rapidly aging population of China, and therefore, reliable early screening and timely monitoring of osteoporosis will be critical for individuals and care providers.

Osteoporosis is diagnosed clinically or radiographically. Biochemical markers of bone turnover in the serum or urine are not currently recommended for diagnosis (Mauck and Clarke, 2006). Bone mineral density (BMD) is a surrogate indicator directly related to bone strength, plays an important role, and is widely used to monitor and diagnose osteoporosis in clinical practice (Engelke, 2012). Currently, dual-energy X-ray absorptiometry (DXA), quantitative computed tomography (QCT), and quantitative ultrasound (QUS) are commonly used tools for evaluating osteoporosis (Malekzadeh et al., 2019). Areal BMD testing *via* DXA in the proximal femur, lumbar spine, and the forearm is the gold standard method for diagnosing osteoporosis, but this does not capture the important contributions of clinical risk factors or other bone measures (e.g., trabecular bone score and geometry) and is susceptible to confounding factors (e.g., osteophyte aortic calcification and body mass index) (Salzmann et al., 2019) (Smets et al., 2021). As defined by the World Health Organization (WHO), for osteoporosis, the DXA BMD criterion requires a T-score of less than −2.5; a normal BMD T-score is higher than −1.0, and osteopenia is anything in-between T-scores −1 and −2.5 (World Health Organization, 1994). Different from areal bone mineral density computed by DXA, BMD derived from QCT is a volumetric measure of the vertebral trabecular bone. Given the high turnover rate of trabecular bone compared to cortical bone (Samelson et al., 2019), BMD calculated from QCT offers substantially higher sensitivity and can also be used for diagnosis based on thresholds published by the American College of Radiology of 120 mg/cc and 80 mg/cc to define osteopenia and osteoporosis, respectively (Cheon et al., 2012). Yet, radiation doses associated with CT and frequent manual operations before QCT image analysis limit the application of QCT in osteoporosis screening.

Quantitative computed tomography can be classified into two main kinds, phantom-based QCT (PB-QCT), which includes synchronously calibrated QCT and asynchronously calibrated QCT, and phantom-less QCT (PL-QCT). The asynchronously calibrated QCT provides results comparable to the established synchronously calibrated QCT. Cheng XG et al. have validated the accuracy and short-term reproducibility of asynchronous QCT and carried out research about asynchronous QCT in population-based clinical studies (Cheng et al., 2014; Wang et al., 2017; Wu et al., 2019). However, the phantom-based QCT needs to deploy a reference calibration phantom during the patient scan, which means the beam hardening and scatter effect cannot be avoided. Although the precision is inferior to phantom-based BMD systems, the mean absolute standardized differences and accuracy deviations between the two methods were small (Habashy et al., 2011; Mueller et al., 2011). PL-QCT has been proved a robust clinical utility for the detection of lowered BMD in a large patient population, which can be easily integrated into the CT workflow for non-dedicated quantitative CT (QCT) BMD measurements in thoracic and abdominal scans and achieved without additional radiation exposure from non-contracted CT scans, to perform an ancillary diagnosis of osteopenia or osteoporosis (Mueller et al., 2011).

Coronavirus disease 2019 (COVID-19) outbreak has rapidly swept around the world, causing a global public health emergency. In diagnosis, chest computed tomography (CT) is used in COVID-19 and is an important complement to the real-time reverse transcription-polymerase chain reaction (RT-PCR) test (Ai et al., 2020). Low-dose chest computed tomography (LDCT), popularly used for early lung cancer screening (National Lung Screening Trial Research Team et al., 2011), can also offer a high specificity for distinguishing COVID-19 from other diseases associated with similar clinical symptoms and has become an indispensable image examination for hospitalized patients in China (Schulze-Hagen et al., 2020). As been confirmed, LDCT can be utilized to measure volumetric bone mineral density (vBMD) (Kim et al., 2017) and shows the feasibility of osteoporotic fracture prevention (Cheng et al., 2021). The combination of LDCT and QCT allows further application of imaging data used for COVID-19 or lung cancer screening to provide an accurate diagnosis of osteoporosis without additional radiation and cost for patients (Pan et al., 2020; Cheng et al., 2021). Cheng XG et al. and Lu Y et al. have validated the efficiency of PB-QCT combined with LDCT through conventional and deep learning methods (Pan et al., 2020; Cheng et al., 2021). Nevertheless, to the best of our knowledge, clinical validation of PL-QCT with LDCT has not been published in a peer-reviewed journal. The purpose of this study was to determine the accuracy and precision of our newly developed automatic PL-QCT system for BMD measurement and osteoporosis assessment for the hospitalized patients in the COVID-19 period based on low-dose chest computed tomography.

## Material and Methods

### Patient Population

**FIGURE 1:**
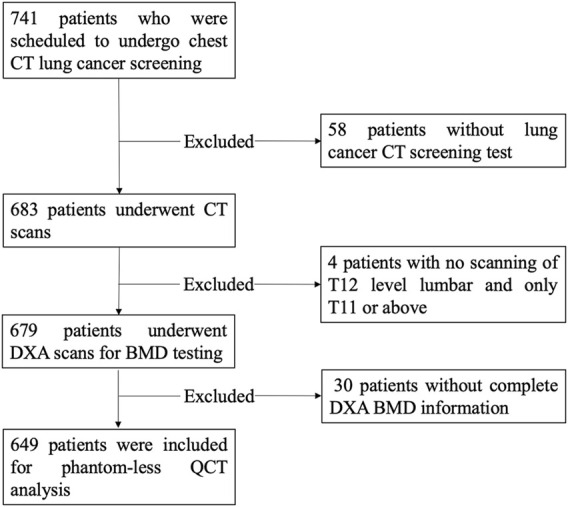
Flow chart of the inclusion process.

The retrospective study was approved by the Institutional Board, informed patient consent was waived, and all information and imaging data were under the control of authors throughout the study. All exams were collected from the patients in The Second Hospital of Jilin University with informed consent and reviewed by the Internal Review Board. A total of 741 patients were scheduled for the DXA and PL-QCT analysis. After the screening process shown in Figure 1, 58 patients were found to have no low-dose CT screening data for lung cancer, and four patients had only T11 and above levels included in the CT image and without T12 level screening. In addition, there were 30 patients whose DXA bone mineral density information was not complete for analysis. A total of 92 patients were excluded, and the remaining 649 patients (Table 1) were included in this study. The average time interval between DXA and QCT scanning of the same patient is 1–3 days.

**TABLE 1:** Basic information of included subjects.

Basic information	Male (*n* = 266)	Female (*n* = 383)	Total subjects (*n* = 649)
Age (years)	55.06 ±12.37	60.02 ±10.47	57.99 ±11.54
Height (cm)	171.80 ±5.71	159.76 ±5.21	164.69 ±8.03
Weight (kg)	76.68 ±12.29	63.74 ±10.08	69.04 ±12.73
BMI ( $\mathrm{kg}/m^{2}$ )	25.96 ±3.82	24.94 ± 3.54	25.36 ±3.69

DXA tests were performed for all patients, including spinal and hip scans and results. At the same time, the newly developed bone density instrument was used to verify. The average DXA BMD results of the total hip and spine were taken as the gold standard. Meanwhile, low-dose chest CT scanning images were used for the analysis and diagnosis by the new phantom-less QCT. The 80 mg/cc and 120 mg/cc were taken as the important criteria for diagnosing osteoporosis and osteopenia in QCT analysis, respectively.

### DXA and CT Acquisition

#### Dual Energy X-Ray Absorptiometry

All patients are performed with DXA on the spine (L1–L4) and hip (femoral neck and total hip). The DXA measurements have been performed on the Hologic device (DXA, Discovery WI, Hologic Inc., USA). The trained technicians and physicians supervised the whole testing process. Since both the spine and hip DXA results were detected, the osteoporosis was diagnosed by the lower T-score of the spine or hip measurement results. According to the international standard, osteoporosis was defined as T-score ≤ −2.5 SD (standard deviation), and osteopenia was defined as −2.5 < T-score  ≤ −1.0 SD.

#### Computed Tomography

The CT images were acquired from several different CT devices, including Philips iCT 256, SCENARIA, NeuViz epoch, and Revolution CT. The scanning parameters of CT are listed in Table 2. These CT images were originally scanned for the lung cancer or COVID-19 screening in the endocrinology department of the hospital.

**TABLE 2:** Low-dose CT scanning parameters.

Manufacturer	NeuViz epoch	Philips-iCT 256	GE-Revolution CT	SCENARIA
Voltage (kV)	120	120	120	120
mA	345	225	254	254
SFOV (mm)	500	500	500	500
Matrix	512*512	512*512	512*512	512*512
Table height (cm)	130.4	150	132.4	122
Slice thickness (mm)	3	1	5	5
Reconstruction kernal	Standard	Standard	Standard	Standard

#### Automatic Phantom-Less QCT BMD Analysis

**FIGURE 2:**
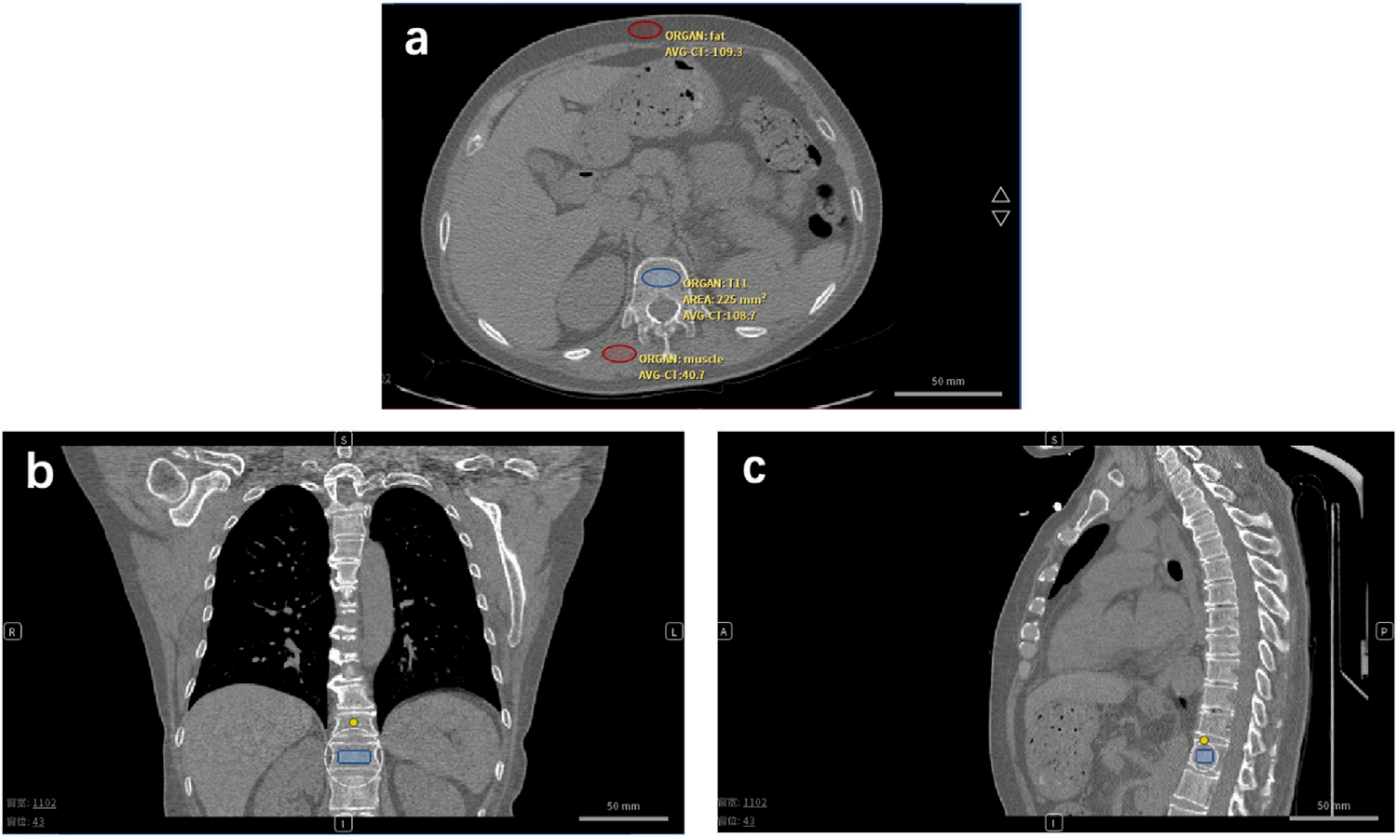
Phantom-less QCT analysis low-dose CT of lung cancer or COVID-19 screening is used for BMD testing and osteoporosis diagnosis. **(A)** Transversal plane, **(B)** coronal plane, and **(C)** sagittal plane of CT image of one enrolled patient. Red ovals represent the muscle and fat ROI. The blue symbol represents the trabecular ROI.

We developed one automatic phantom-less QCT software, which can be applied in the spine and hip BMD measurements. This novel PL-QCT has the automatic function of selecting the vertebrae, hip, fat, and muscle ROI and calibrating the BMD with high precision. A detailed phantom-less QCT technology development process can be found in our last study (Liu et al., 2021). Fat and muscle ROI CT values have been used to calibrate the BMD results (Figure 2). Localized BMD can also be accurately measured, including cancellous and cortical bone. Compared with phantom-based QCT, phantom-less QCT can be utilized to measure BMD without simultaneous scanning of the external phantom. There were many reports on the phantom-less QCT development and relative bone mineral density of fat and muscle.

### Statistical Analysis

#### Osteoporosis Analysis Results by DXA and QCT

Consistency analysis was performed on the BMD results of DXA and QCT. The diagnosis rates of osteoporosis, osteopenia, and normally detected by DXA and QCT were compared and analyzed. Receiver operating characteristic curve (ROC) analysis and confusion matrix analysis were conducted, respectively. The results calculated by DXA were used as the gold standard for the diagnosis of osteopenia and osteoporosis. The diagnostic efficacy of QCT in female and male subgroups was also analyzed by ROC (area under curve: AUC value).

#### BMD Changes With Age

**FIGURE 3:**
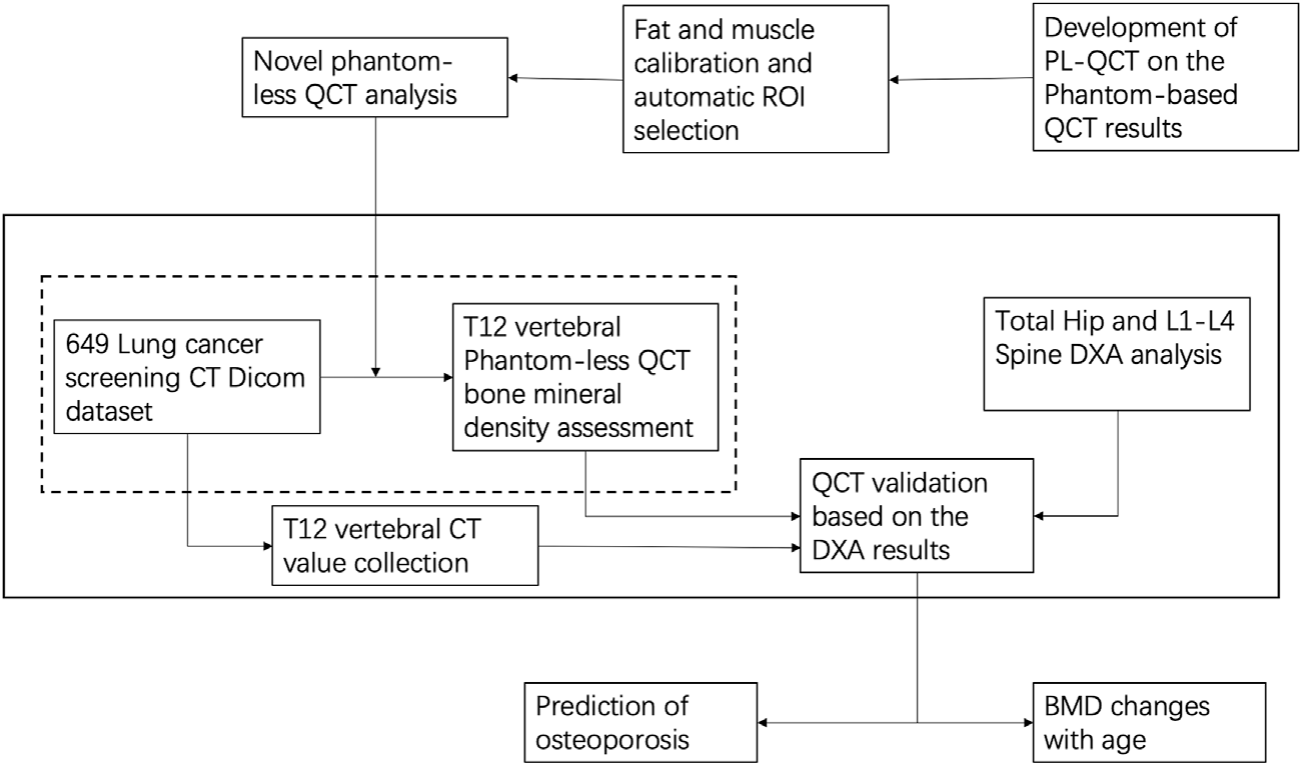
Flow chart of the testing process in the whole project.

The enrolled patients were divided into seven subgroups by age. The mean value and standard deviation of different subgroups were calculated, respectively, and the correlation between the DXA and phantom-less QCT methods was analyzed. The whole research step is shown in Figure 3.

#### BMD Measured by Different CT Devices

**FIGURE 4:**
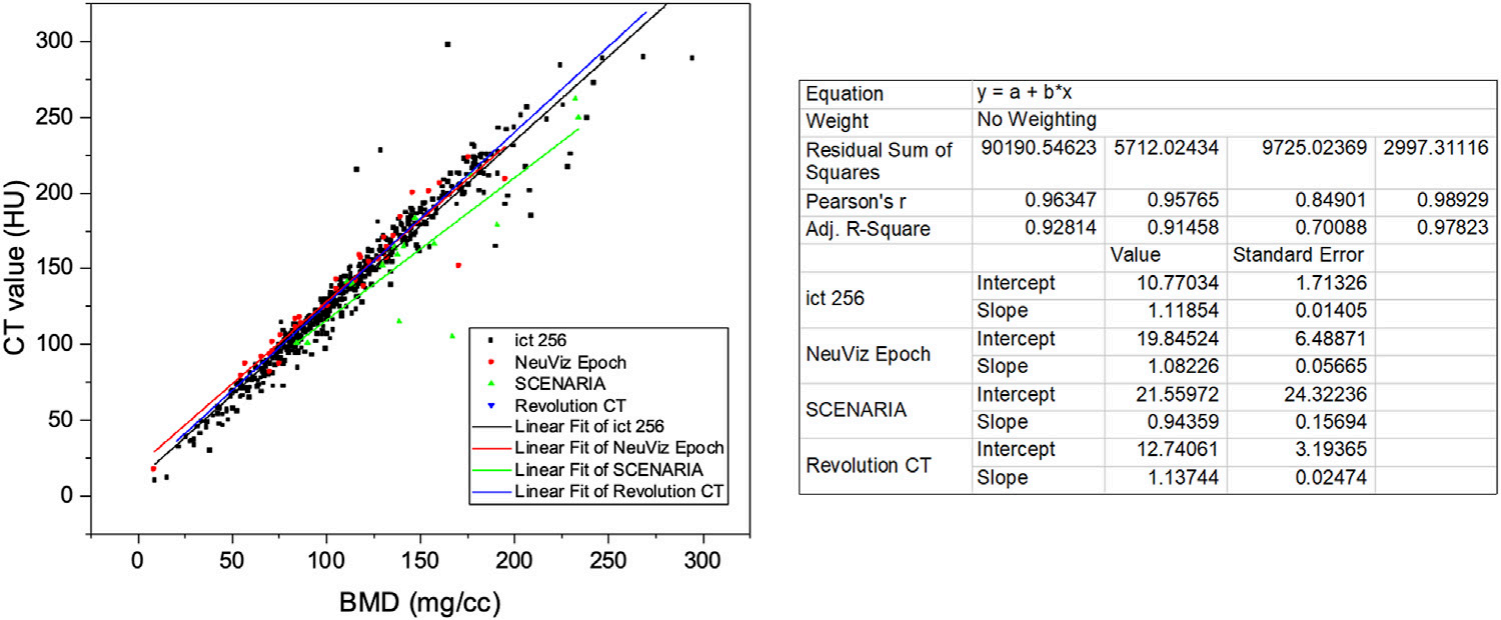
Linear relationship between CT attenuation values and vBMD (mg/cc) of different CT devices.

The patients were scanned by four main types of CT devices. In some studies, Hounsfield unit (HU) values were used to represent BMD and diagnose osteoporosis. To investigate the influence of the CT devices on the HU value, we have studied the relationship between the CT value and BMD calculated by phantom-less QCT for different CT devices (Table 3 and Figure 4).

**TABLE 3:** Comparison between the precision of different QCT studies (Liu et al., 2021).

Result and reference	Phantom-less QCT result			Phantom-based QCT result
	Automatic PL-QCT	Philips	Other study	Mindways
Precision in SD[ $\text{mg}/{\text{cm}}^{3}$ ]	0.87	3.1	—	—
Precision as CV[%]	0.89	4.0	1–2	1.4–3.6

## Results

### Patient Population

After the patient enrollment screening, the data of 649 patients meeting the conditions were retained for validation analysis, and the basic information of patients was collected. The average age of the whole cohort of patients is 57.99 ( ± 11.54) years. The height is 164.69 ( ±8.03 ) cm, and the weight is 69.04 ( ±12.73 ) kg. The body mass index (BMI) of these patients is 25.36 ( ± 3.69) kg/m^2^.

### Comparison Between the Diagnosis Rate of Osteoporosis and Osteopenia of DXA and QCT

**FIGURE 5:**
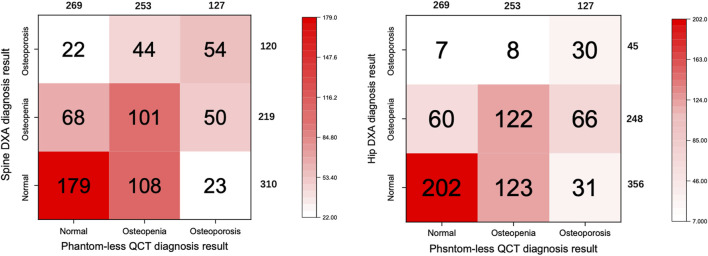
Confusion matrix of QCT and spine DXA diagnosis result comparison.

**FIGURE 6:**
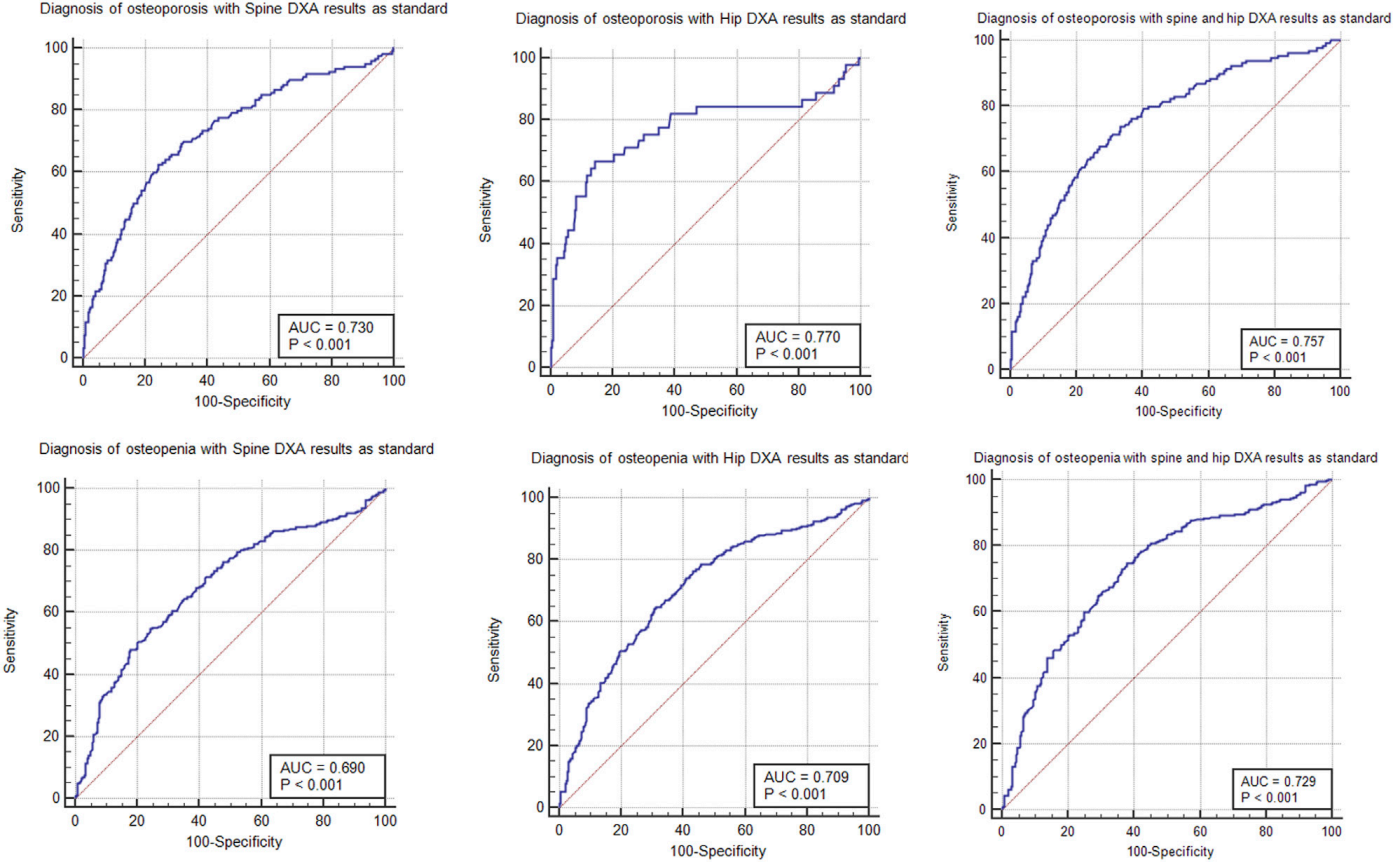
ROC analysis of PL-QCT and DXA diagnosis result comparison.

The different diagnosis rates of osteoporosis, osteopenia, and normal patients for spine DXA result, hip DXA result, and phantom-less QCT results are shown in Figure 5. Hip and spine DXA results have been, respectively, settled as the golden standards for the analysis of QCT. Due to surgeons using the lower value of the hip and spine DXA result to diagnose osteoporosis in clinical practice, we also set this lower value as another reference in the ROC analysis (Table 4). According to the results of ROC analysis, the AUC index basically remained above 0.7, indicating that bone mineral density calculated by phantom-less QCT can predict bone loss and osteoporosis. However, the BMD results measured by DXA are often higher due to vascular calcification and osteophytes. This leads to a relatively higher false-negative rate in diagnosing osteoporosis for DXA. Thus, a difference exists between the diagnosis rates of the two methods (as shown in Figure 6), and this can partly explain why the AUC values in the ROC analysis are not so high. In this study, we aim to explore the clinical application potential of the automatic phantom-less QCT, and the results in Figure 5 and Figure 6 are able to demonstrate the effectiveness of the new method to some extent, but further validation involving comparison with other accurate devices still needs to be conducted.

**TABLE 4:** ROC analysis of QCT results with spine and hip DXA as the golden standard.

		Diagnosis	AUC (95%CI)	Sensitivity (%)	Specificity (%)	Youden index J	Associated criterion
Hip DXA result	Women (*n* = 383)	Osteoporosis	0.74 (0.69–0.78)	67.5	81.9	0.49	≤77.8
		Osteopenia	0.71 (0.66–0.75)	67.1	68.2	0.35	≤105.9
	Men (*n* = 266)	Osteoporosis	0.88 (0.84–0.92)	100	68.2	0.68	≤102.3
		Osteopenia	0.68 (0.62–0.74)	82.5	51.1	0.36	≤129.0
	Total (*n* = 649)	Osteoporosis	0.77 (0.74–0.80)	66.7	85.8	0.52	≤77.8
		Osteopenia	0.71 (0.67–0.74)	64.5	69.1	0.34	≤106.1
Spine DXA result	Women (*n* = 383)	Osteoporosis	0.72 (0.67–0.76)	68.7	69.0	0.38	≤97.2
		Osteopenia	0.72 (0.67–0.76)	58.0	82.1	0.40	≤98.4
	Men (*n* = 266)	Osteoporosis	0.71 (0.66–0.77)	76.2	65.3	0.42	≤107.2
		Osteopenia	0.63 (0.57–0.69)	77.2	49.7	0.27	≤130.7
	Total (*n* = 649)	Osteoporosis	0.73 (0.69–0.76)	62.5	75.6	0.38	≤92.5
		Osteopenia	0.69 (0.65–0.73)	54.9	75.8	0.31	≤101
Lower value of spine and hip DXA result	Women (*n* = 383)	Osteoporosis	0.74 (0.69–0.78)	70.1	69.9	0.40	≤97.2
		Osteopenia	0.74 (0.69–0.78)	68.4	71.2	0.40	≤112.4
	Men (*n* = 266)	Osteoporosis	0.76 (0.71–0.81)	81.0	66.9	0.48	≤107.2
		Osteopenia	0.70 (0.64–0.75)	78.7	58.3	0.37	≤130.7
	Total (*n* = 649)	Osteoporosis	0.76 (0.71–0.79)	66.2	74.6	0.41	≤95.6
		Osteopenia	0.73 (0.69–0.76)	74.7	62.0	0.37	≤122.2

### BMD Changes Are Associated With Age for Males and Females

**FIGURE 7:**
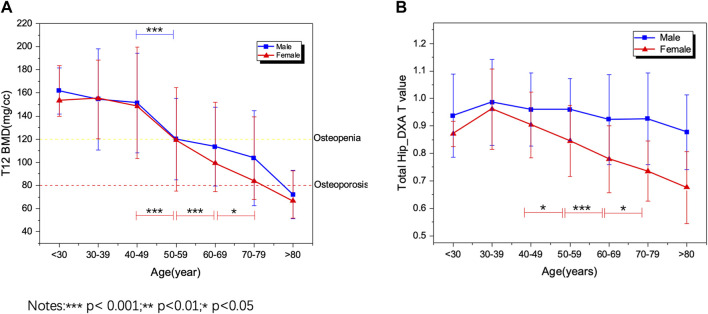
PL-QCT and DXA BMD changes with age. **(A)** PL-QCT; **(B)** DXA.

The BMD changes have been measured by the QCT and DXA results, relatively absolute BMD value, and T-score of the DXA. The result in Figure 7 shows that BMD decreases significantly after 40–49 years old, especially for female patients. This result is similar to other studies (Cheng et al., 2021). However, no study has utilized the phantom-less QCT to do the large data screening based on the lung cancer or COVID-19 screening LDCT images. From the DXA BMD results, the change of T-score in the female group has a similar trend (Figure 7), but the male groups have a large difference among the spine DXA, femoral neck DXA, and total hip DXA (Supplementary Figures S3).

### BMD Measured by Different CT Devices

Four main CT devices were used for CT scanning in this study. CT values are correlated with BMD values, but different CT devices and scanning parameters have an impact on the specific relationship between CT and BMD. Therefore, CT values cannot be directly used as a diagnostic method of osteoporosis in clinical applications. It can be found from the results that the linear regression relationship between CT and BMD is not exactly the same for analysis in different CT images scanned by different CT machines.

## Discussion

In this study, we determined the accuracy and precision of our newly developed automatic PL-QCT in BMD measurement and osteoporosis detection based on the retrospectively collected LOCCT scans for COVID-19 diagnosis, lung cancer, or other indications. To our knowledge, it is the first time for automatic PL-QCT to evaluate the performance against DXA in LDCT images.

Sensitive detection of bone mineral density (BMD) change is a key issue in monitoring and evaluating the individual bone health status, as well as bone metabolism and bone mineral status. Matthew J Budoff et al. have validated that the thoracic and the lumbar QCT provides a similar and much sensitive method for detecting bone mineral loss when compared to DXA (Mao et al., 2017). The accuracy and short-term reproducibility of asynchronous PB-QCT have been verified in a nationwide multicenter study carried out by Cheng et al. (2014), Wang et al. (2017), and Wu et al. (2019), and the lumbar CT has been recommended as analogous to central DXA technologies in assessing or monitoring ages and disease- and treatment-related BMD changes in PB-QCT. PL-QCT does not need to deploy a reference calibration phantom during the patient scan compared with PB-QCT, which uses surrounding tissue like fat and muscle as calibration so that the beam hardening and scatter effect can be avoided (Budoff et al., 2013; Michalski et al., 2020). Nevertheless, conventional QCT analysis, whichever the phantom-based or -less, requires manual localization of vertebral bodies and region of interest (ROI) (National Lung Screening Trial Research Team et al., 2011). Hence, it is necessary to develop an automatic QCT to localize vertebral bodies and select suitable fat or muscle ROI, as well as calculate bone density with high precision. Lu Yet al. developed useful automatic QCT image analysis software based on the deep learning method in LDCT images, which eliminate the heavy manual operation in BMD measurement and liberate the radiologist from reduplicative tasks (Pan et al., 2020). In a previous study, our group also developed an automatic phantom-less QCT system based on traditional machine learning methods in lumbar CT images, which shows high BMD measurement precision with the automatic selection of fat and muscle ROI (Liu et al., 2021). In this study, we further validated the capability and precision of our automatic PL-QCT system in LDCT so as to enhance its possibility of being integrated into the CT workflow in large-scale osteoporosis screening.

DXA is the most common method for the estimation of BMD and fracture risk in the clinical setting. Therefore, the DXA spine and hip BMD standards were utilized as the reference in the diagnosis rate and ROC analysis of the comparison between DXA and PL-QCT. According to the results of ROC analysis, the average AUC index basically remained above 0.75, especially in the situation of the lower value of the hip and spine DXA, indicating that bone mineral density calculated by phantom-less QCT can predict bone loss and osteoporosis. Compared to DXA, the automatic PL-QCT detected a relatively higher proportion of osteoporosis patients, and this may be due to the false-negative cases caused by the osteophyte and vascular calcification in DXA diagnosis. Many studies have also reported similar results regarding the comparison between DXA and QCT (Li et al., 2013). The associated criterion is that BMD is less than 77.8 mg/cc and 92.5 mg/cc in the hip and spine DXA result group, respectively, for this automatic PL-QCT system, which is different from the common standard of 80 mg/cc. Several studies have shown that BMD is higher in the thoracic spine than the lumbar spine (Weishaupt et al., 2001). Due to the low sensitivity of DXA, some patients with osteoporosis may be misjudged, especially the elderly, and may not receive timely treatment, which increases the risk of osteoporotic fractures. Therefore, the current clinical guidelines do not recommend DXA for screening in the United Kingdom, which also explains the relatively lower sensitivity, specificity, and Youden index of this PL-QCT.

After validating the potential function of this PL-QCT in distinguishing osteoporosis and measuring BMD, we also measured the mean and S.D. of BMD variation with age by QCT and compared the trend measured by DXA. Figure 3 shows the age-dependent mean vBMD for each 10-year interval. Thoracic spine BMD was decreased progressively with age, varying in women from 155.19 mg/cc at age 30–39 years to 66.59 mg/cc at age 80+ years and in men from 161.7 to 72.2 mg/cc. There was a greater rate of bone loss in women than men after the age of 49 years, suggesting the influence of menopause on bone loss. All these results and the tendency are similar to the lumbar spine or low-dose chest CT measured by PB-QCT (Ghildiyal et al., 2018; Cheng et al., 2021). The reliability and accuracy of HU to BMD measurement and determining osteoporosis have been proven in the literature with many reports (Lee et al., 2013; Park et al., 2020), but in its current state, it is not ready for clinical implementation. There is a lack of exchangeability among different machines that limits its broad applicability (Gausden et al., 2017). In our study, we included four main CT devices for BMD measurement, and it can be found that the results between CT value and BMD are not exactly the same for analysis in different CT images scanned by CT machines. However, the similar linear regression relationship between these four machines indirectly indicates the robustness of our PL-QCT.

There were a few limitations to this study. First, the retrospective study used DXA of the lumbar spine instead of the QCT, which could provide a more reliable evaluation of the performance of our developed system as a reference standard for BMD measurement. It is difficult to find any individuals who underwent LDCT and QCT within a short time, which may cause more radiation and high cost. Second, all LDCT scans were obtained at a single center in this study. Further confirmation of the consistency, robustness, and transferability of this system in LDCT scans using scanners from multi-center institutions will be implemented.

## Conclusion

In order to achieve fully automated BMD measurement and osteoporosis detection on LDCT scans, a newly automatic PL-QCT system was developed in company with auto-location and detection function-based traditional machine learning methods. The performance of the system was evaluated by using DXA as the reference standard. To our knowledge, it is the first time for automatic PL-QCT to evaluate the performance against DXA in LDCT images. The accuracy and precision of the system for BMD measurement and osteoporosis indicate that it may be a promising tool for individuals screened for low-dose chest computed tomography.

## Data Availability

The datasets presented in this article are not readily available because the data presented in this study are available on request from the corresponding author. The data are not publicly available due to the restriction of IRB. Requests to access the datasets should be directed to qinyg@jlu.edu.cn.
