# Supplementary material for: One Novel Phantom-Less Quantitative Computed Tomography System for Auto-Diagnosis of Osteoporosis Utilizes Low-Dose Chest Computed Tomography Obtained for COVID-19 Screening
Source: Front Bioeng Biotechnol. 2022 Jun 28;10:856753. doi: 10.3389/fbioe.2022.856753 (PMC9273929; doi:10.3389/fbioe.2022.856753)

Supplementary Figures 1. ROC analysis of PL-QCT and DXA diagnosis result comparison of subgroup of Male

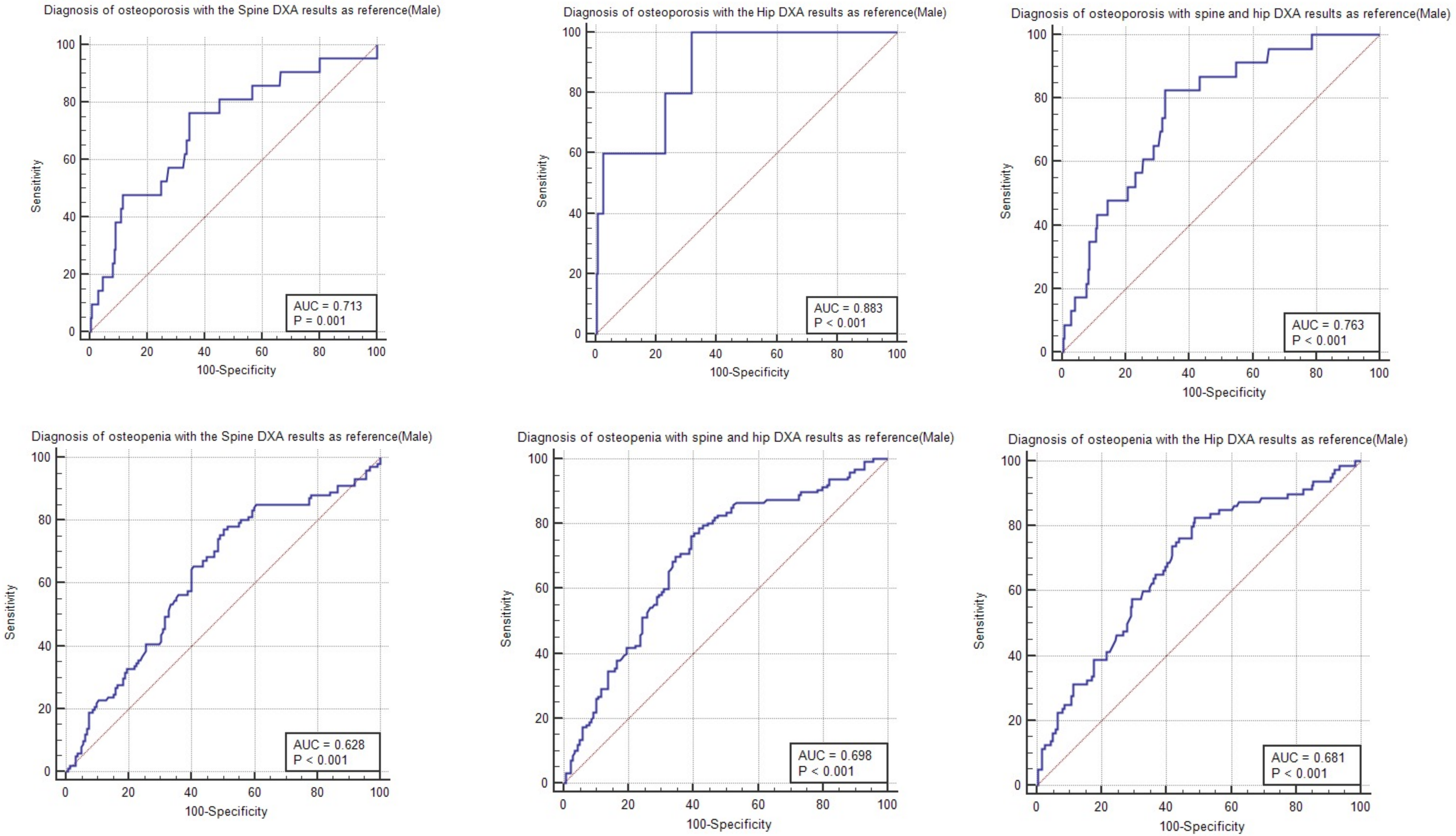

Supplementary Figures 2. ROC analysis of PL-QCT and DXA diagnosis result comparison of subgroup of Female

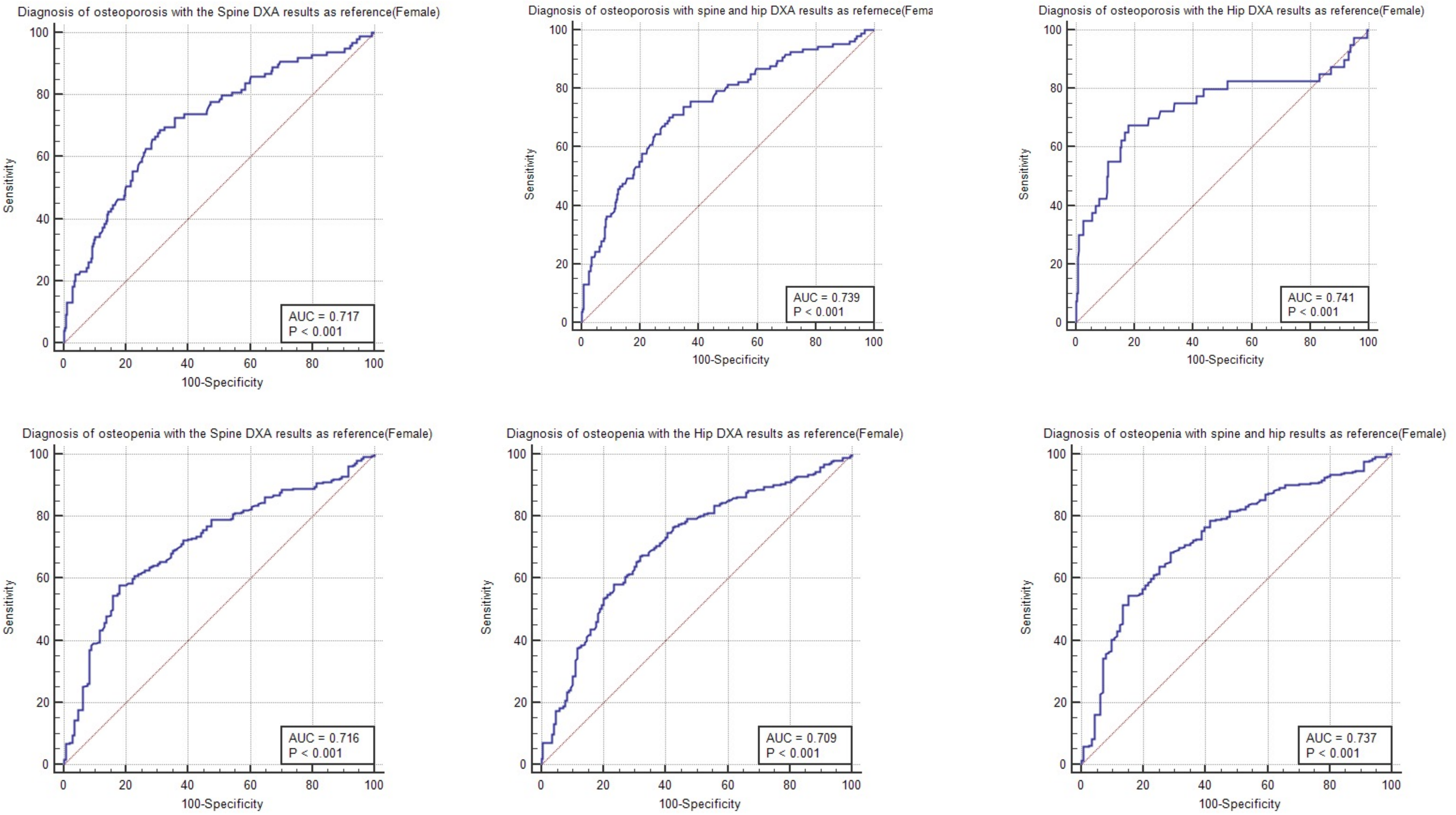

Supplementary Figures 3. Femal Neck and Spine DXA BMD changes with age

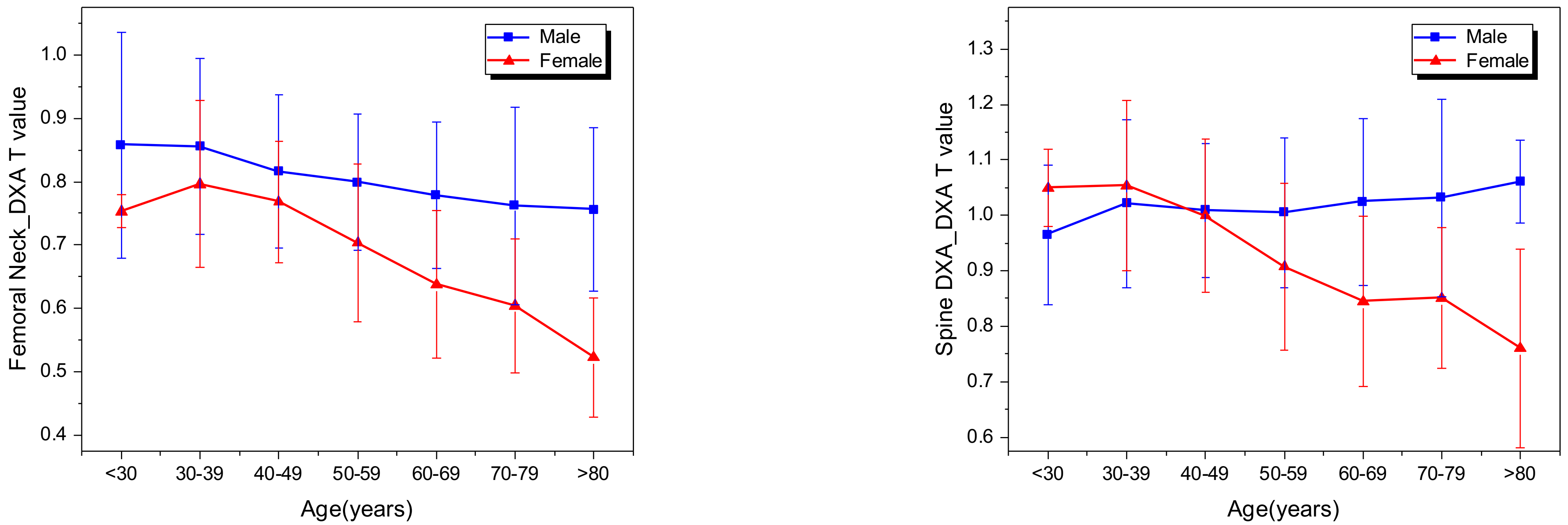

Supplement: Supplementary file 1 [file DataSheet1.PDF]
